# DFNA5 regulates immune cells infiltration and exhaustion

**DOI:** 10.1186/s12935-022-02487-0

**Published:** 2022-03-05

**Authors:** Jian Hu, Wenceng Pei, Minren Jiang, Ying Huang, Fuyun Dong, Zhenyou Jiang, Ying Xu, Zihuang Li

**Affiliations:** 1 Department of Oncology Radiotherapy, The Second Clinical Medical College, Jinan University (Shenzhen People’s Hospital), Shenzhen, 518020 China; 2 Department of Gastroenterology, Civil Aviation Hospital of Shanghai, Shanghai, China; 3 Department of Gastroenterology, Ruijin Hospital of Shanghai, Shanghai, China; 4 Shanghai Fifth People’s Hospital, Fudan University, Shanghai, China

**Keywords:** DFNA5, Tumour-infiltrating lymphocytes, Prognosis, Cancer, CD274

## Abstract

**Background:**

DFNA5 (GSDME) belongs to Gasdermin familily that is involved in a variety of cancers and triggers cell pyroptosis after chemical treatment. However, the relationship in DFNA5 between prognosis and immune cells in diverse cancers has been receiving little attention. Tumor immune cells infiltration and exhaustion may associate with patients prognosis. The roles of DFNA5 in tumor immune cells infiltration and exhaustion have not been clarified.

**Methods:**

The expression level of DFNA5 was determined by the Tumour Immune Estimation Resource and the Oncomine database. Then the impacts of DFNA5 in prognosis were assessed by Kaplan–Meier plotter and ULACAN. The correlations between DFNA5 and tumour-infiltrating lymphocytes were explored by TIMER. In addition, the relationships in the expression levels of DFNA5 and typical genes combination of tumour-infiltrating lymphocytes were analysed by GEPIA and TIMER. In this study, we screened the chemokine and immune related proteins interacted with DFNA5 using TurboID system to explore the instantaneous or weak interactions.

**Results:**

DFNA5 significantly influences the prognosis in different cancers according to The Cancer Genome Atlas (TCGA). The expression levels of DFNA5 showed positive correlations to the infiltration of macrophages, CD8 + T cells, CD4 + T cells in liver hepatocellular carcinoma (LIHC), colon adenocarcinoma (COAD), and lung adenocarcinoma (LUAD). DFNA5 expression displayed obvious correlations with multiple lymphocytes gene makers in COAD, LIHC and LUAD. DFNA5 expression has effects on the prognosis of liver hepatocellular carcinoma and LUAD. DFNA5 upregulated the expression levels of PDCD1 and CD274 in a dose-dependent manner. Chemokine and immune related proteins interact with DFNA5.

**Conclusions:**

These results indicate that DFNA5 is related to patient prognosis and immune cells, consisting of macrophages, CD4 + T cells, and CD8 + T cells, in diverse cancers. In addition, DFNA5 expression might contribute to the regulation of T cell exhaustion, tumour-associated macrophages (TAMs), and Tregs in COAD, LIHC and LUAD. DFNA5 may regulate immune infiltration via EIF2AK2. IFNGR1 was related to the functions of PD-L1 expression and PD-1 checkpoint pathway. These results indicate that DFNA5 levels may be act as a prognostic factor and predict the degrees of immune cells infiltration in LIHC and LUAD.

**Supplementary Information:**

The online version contains supplementary material available at 10.1186/s12935-022-02487-0.

## Introduction

Conlon, liver, lung cancers are the common malignancies in most countries, and immune cells infiltration into tumour tissue and immune cells exhaustion lead to a poor prognosis [1–4]. The infiltration number and exhaustion state of immune cells could exert a key role in cancer progression, and immune combination therapy is becoming a promising strategy for enhancing traditional chemotherapy. At present, the most popular immune checkpoint inhibitors are programmed death-1 (PD-1), programmed death ligand-1 (PD-L1) and cytotoxic T lymphocyte associated antigen 4 (CTLA4), which are promising antitumour therapies in multiple cancers [5–7]. However, the efficacy of immunotherapies, including anti-CTLA4, anti-PD-L1 and anti-PD-1, differ from person to person. Furthermore, the study of tumour-infiltrating lymphocytes has become a hotspot, and tumour-associated macrophages (TAMs), CD8 + T cells and CD4 + T cells have effects on patient prognosis and are correlated with the efficacy of immunotherapy and chemotherapy in the clinic [7–9]. Therefore, there is an urgent need to explore the recruitment types of immune cells and to discover potential targets associated with a better prognosis.

Cell death is the basic processes in the cellular life activities, which maintains homeostasis along with cell proliferation, differentiation in multicellular organisms. Apoptosis, necrosis and pyroptosis are the common cell death forms. Apoptosis is a type of programmed cell death characteristics of intact cell membrane and generally not inducing inflammation. Necrosis is a passive type of cell death caused by pathological stimuli. Similar to apoptosis, pyroptotic cells undergo nuclear condensation and chromatin DNA fragmentation, and TUNEL staining is positive. Similar to necrosis, the membrane pores formation can lead cell swelling, cell membrane rupture, and release of inflammatory factors consisting of LDH, HMGB1, IL- 1β, IL-18. Pyroptosis is mediated by the gasdermin family. The relationship between pyroptosis and tumours differs in different tissues and genetic backgrounds. It can inhibit the occurrence and development of tumours. On the other side, it can promote tumour growth due to release of proinflammatory factors. Studies have shown that DFNA5 methylation results in lower expression levels of DFNA5 in most tumour cells than in normal cells. Specific drugs can be chosen to upregulate the expression levels of DFNA5 to enhance anti-tumour effects. The pyroptosis may play key role in the antitumour efficacy.

Previous reports suggested that DFNA5 is a gene associated with deafness [10]. Recently, DFNA5 was discovered to play an important role in pyroptosis [11]. DFNA5 is cleaved by caspase3 after drug treatment. The N-terminal part of DFNA5 can create pores in the cell membrane, and then the cellular content can be released outside [12–14]. In the tumour microenvironment, cellular components, such as cytokines, may recruit immune cells into tumour tissue to exert antitumour efficacy [15]. Although DFNA5 is usually recognized as a tumour suppressor gene, its expression levels in diverse cancers are different. However, the underlying mechanisms of DFNA5 in tumour immunology are still unclear.

In this research, we explored the relationship of DFNA5 expression levels to diverse cancer patients based on the databases including Kaplan–Meier plotter, UALCAN, Oncomine. Furthermore, Tumour Immune Estimation Resource was used to investigate the relationship between DFNA5 levels and lymphocytes in diverse cancer. The analyses in this study illustrate the importance of DFNA5 in Conlon, liver, lung cancers and its role in lymphocytes infiltration and T cell exhaustion.

## Materials and methods

### Cells and cell culture

Human embryonic kidney 293T cells (HEK-293T), obtained from American Type Culture Collection, were cultured in Dulbecco’s modifified Eagle medium (Invitrogen) containing 10 v/v % fetal bovine serum (FBS, Gibco fetal bovine serum) and 1 v/v % penicillin/streptomycin (Sigma-Aldrich) at 37 °C in an atmosphere of 5% CO2.

### Expression vector construction

A pcDNA3.0-flag, pcDNA3.0-PDCD1-3x-myc and pcDNA3.0-CD274-3x-myc plasmid expression vector were obtained from our lab. A pcDNA3-based expression vector for TurboID was obtained from addgene(Addgene: #107169). PLVX-Puro-DFNA5 was obtained from addgene (Addgene: #154876). A pcDNA3.0-DFNA5-flag expression vector was constructed using PLVX-Puro-DFNA5 as a template. We used overlapping RCR method to link DFNA5 gene and TurboID together (DFNA5-TurboID). Then DFNA5-TurboID was recombined into pcDNA3.0-flag vector using ClonExpress Ultra One Step Cloning Kit (Vazyme, C115-01).

### Flag-DFNA5 plasmids co-transfected with PDCD1-3xmyc or CD274–3xmyc plasmid

DFNA5 could influence the expression of PDCD1 and CD274. 293 T Cells were seeded into 6 well plate for 24 h. The increased content of Flag-DFNA5 plasmid co-transfected with PDCD1-3xmyc or CD274-3xmyc plasmid for 48 h, then the cells protein was extracted by RIPA lysis buffer. RIPA lysis buffer was obtained from Beyotime Institute of Biotechnology.

### DFNA5 could upregulate the expression levels of PDCD and CD274 (Western blot)

Monoclonal antibodies against β-actin-HRP were purchased from Proteintech. Monoclonal antibodies of Myc and FLAG was purchased from Cell Signalling Technology. The expression levels of Flag-DFNA5, CD274-3xmyc, PDCD1-3xmyc, β-actin were detected by Western blot. Protein was transferred to a PVDF membrane. After blocking with 5% nonfat milk, the membrane was incubated with Monoclonal antibodies at 4℃ overnight, followed by incubation with HRP-labeled secondary antibodies for 1 h. The protein bands were exposured by imaging system (Tangen).

### DFNA5-TurboID expression vector construction for screening Instantaneous interaction proteins

TurboID system is used to identify the instantaneous or weak interaction proteins. TurboID and DFNA5-TurboID were transfected into 293 T cells, respectively. The cells were cultured for 48 h after transfection, then biotin was added to the culture medium for 10 min. TurboID was used as a control to exclude the binding itself or nonspecific binding. Then RIPA lysis buffer was used to extract the protein. Streptavidin beads were used to pull the interactive proteins. And then the proteins were identified by mass spectrometry.

### Oncomine database analysis

The gene expression level of DFNA5 in diverse cancers is indicated in the Oncomine database (https://www.oncomine.org/resource/login.html). The threshold was identified based on values: fold change of P-value of 0.001, 1.5, and all genes are ranked [16].

### Kaplan–Meier plotter database analysis

We used Kaplan–Meier plotter to assess the effect of 54 k genes on survival according to 14,912 cancer samples (http://kmplot.com/analysis/) [17]. The data included 1440 gastric, 2190 ovarian, 7830 breast, and 3452 lung cancer samples. The relationship of DFNA5 expression to survival in gastric cancers, ovarian cancer, breast cancer, lung cancer, and liver cancer was evaluated by Kaplan–Meier plotter. Log-rank P-values and hazard ratios (HRs) with 95% confidence intervals were also determined.

### TIMER database analysis

TIMER is a combined database for analysing immune cell infiltration in tumour sites of multiple cancer types (https://cistrome.shinyapps.io/timer/) [18]. The abundance of tumour lymphocytes from gene expression profiles was deduced by the deconvolution method in TIMER based on a previous report [19].

The TIMER database consists of 10,897 samples from 32 cancer types for evaluating the levels of immune cell infiltration. DFNA5 expression was analysed in multiple cancer types to investigate the relationship between DFNA5 expression and the levels of immune cell infiltration, including macrophages, dendritic cells, B cells, CD4 + T cells, CD8 + T cells, and neutrophils. Gene expression levels correlated with immune cell infiltration are shown in Table 1. Furthermore, we explored the relationship between gene markers of immune cells and DFNA5 expression by correlation analysis. The gene markers of the immune cells consist of monocytes, B cells, CD8 + T cells, T cells (general), M1 macrophages, M2 macrophages, TAMs, neutrophils, Tregs and exhausted T cells according to previous studies. DFNA5 was used with gene symbols on the x-axis and related marker genes of gene symbols on the y-axis. The gene expression level was represented as log2 RSEM.

**Table 1:** Correlation analysis between DFNA5 and relate genes and markers of immune cells in TIMER

Desription	Gene markers	COAD				LIHC				LUAD			
		None		Purity		None		Purity		None		Purity	
		Cor.	P	Cor.	P	Cor.	P	Cor.	P	Cor.	P	Cor.	P
CD8 + T cell	CD8A	0.37	***	0.267	***	0.182	***	0.138	0.0102	0.196	***	0.103	0.0226
	CD8B	0.302	***	0.254	***	0.165	***	0.125	0.0201	0.172	***	0.104	0.0206
T cell (general)	CD3D	0.35	***	0.223	***	0.234	***	0.198	***	0.226	***	0.119	*
	CD3E	0.426	***	0.323	***	0.213	***	0.174	*	0.252	***	0.144	*
	CD2	0.417	***	0.32	***	0.216	***	0.184	***	0.263	***	0.16	***
B cell	CD19	0.255	***	0.133	*	0.191	***	0.139	*	0.112	*	0.002	***
	CD79A	0.318	***	0.18	***	0.208	***	0.165	*	0.123	*	0.025	0.579
Monocyte	CD86	0.706	***	0.659	***	0.33	***	0.319	***	0.496	***	0.45	***
	CD115 (CSF1R)	0.711	***	0.662	***	0.265	***	0.256	***	0.497	***	0.442	***
TAM	CCL2	0.67	***	0.623	***	0.242	***	0.203	***	0.439	***	0.391	***
	CD68	0.543	***	0.489	***	0.329	***	0.296	***	0.462	***	0.412	***
	IL10	0.546	***	0.516	***	0.31	***	0.291	***	0.375	***	0.303	***
M1 Macrophage	INOS (NOS2)	− 0.153	***	− 0.208	***	0.0036	0.486	0.032	0.557	0.156	***	0.117	*
	IRF5	0.366	***	0.387	***	0.196	***	0,193	***	0.359	***	0.309	***
	COX2(PTGS2)	0.255	***	0.196	***	0.236	***	0.213	***	0.164	***	0.17	***
M2 Macrophage	CD163	0.725	***	0.683	***	0.194	***	0.17	*	0.427	***	0.375	***
	VSIG4	0.713	***	0.667	***	0.284	***	0.261	***	0.431	***	0.39	***
	MS4A4A	0.697	***	0.658	***	0.225	***	0.202	***	0.433	***	0.383	***
Neutrophils	CD66b (CEACAM8)	− 0.101	*	− 0.0074	0.136	0.191	***	0.193	***	0.098	0.0267	0.079	0.0787
	CD11b (ITGAM)	0.0737	***	0.0717	***	0.327	***	0.328	***	0.496	***	0.453	***
	CCR7	0.4	***	0.3	***	0.197	***	0.18	***	0.245	***	0.134	*
Treg	FOXP3	0.551	***	0.487	***	0.188	***	0.197	***	0.197	***	0.26	***
	CCR8	0.562	***	0.51	***	0.315	***	0.302	***	0.283	***	0.208	***
	STAT5B	0.364	***	0.369	***	0.167	*	0.198	***	0.18	***	0.153	***
	TGFβ	0.698	***	0.624	***	0.291	***	0.268	***	0.474	***	0.419	***
T cell exhaustion	CD274	0.445	***	0.37	***	0.192	***	0.189	***	0.384	***	0.312	***
	PDCD1	0.386	***	0.294	***	0.208	***	0.169	*	0.297	***	0.19	***
	CTLA4	0.469	***	0.39	***	0.25	***	0.224	***	0.259	***	0.157	***
	LAG3	0.353	***	0.252	***	0.161	*	0.136	***	0.218	***	0.144	*
	TIM-3 (HAVCR2)	0.731	***	0.696	***	0.358	***	0.359	***	0.495	***	0.449	***

*COAD* colon adenocarcinoma, *LIHC* liver hepatocellular carcinoma, *LUAD* lung adenocarcinoma, *TAM* tumor-associated macrophage, *Cor* R value of Spearman’s correlation; Purity, correlation adjusted by purity. None, correlation without adjustment.*P < 0.01; **P < 0.001; ***P < 0.0001

### UALCAN database analysis

UALCAN is a comprehensive web resource for analysing cancer (TCGA, MET500 and CPTAC). It is built on PERL-CGI with high-quality graphics based on JavaScript and CSS. UALCAN provides patient survival information for protein-coding genes with easy access to publicly available data (http://ualcan.path.uab.edu/) [20].

### Gene correlation analysis in GEPIA

The online database Gene Expression Profiling Interactive Analysis (GEPIA) was used to further confirm the significantly correlated genes in TIMER. GEPIA is an interactive website from TCGA and the GTEx projects that analyses RNA sequencing expression (http://gepia.cancer-pku.cn/index.html) [21]. Gene expression correlation analysis was conducted for the given sets of TCGA expression data by the Spearman method. DFNA5 is represented on the x-axis, and related genes are displayed on the y-axis.

### DAVID

TurboID system can explore the instantaneous or weak interaction molecules. In this study, we screened the chemokine and immune related Genes interacted with DFNA5 using TurboID system. Then the screened proteins were analyzed by DAVID(https://david.ncifcrf.gov/) [22]. Gene ontology analyses focus on three domains: biological processes (BP), cellular components (CC), and molecular functions (MF), and such analyses are commonly used to predict the functional roles of chemokine and immune related Genes interacted with DFNA5, while KEGG analysis can define the pathways related to the DFNA5 associated with immune function.

### Statistical analysis

Survival curves were provided by Kaplan–Meier plots and UALCAN. The figures obtained from Oncomine are presented with fold changes, ranks and P-values. The results of GEPIA and Kaplan–Meier plots are presented with Cox P, P or HR values from a log-rank test. Spearman’s correlation and statistical significance are typical standards to assess the correlation of gene expression. The correlative strength was determined according to the following: 0.70–1.0 “strong”, 0.40–0.70 “moderate”, 0.10–0.40 “weak”, and 0.00–0.10 “very weak”. P-values < 0.05 were considered statistically significant.

## Results

### The mRNA expression levels of DFNA5 in different types of human cancers

**Fig. 1:**
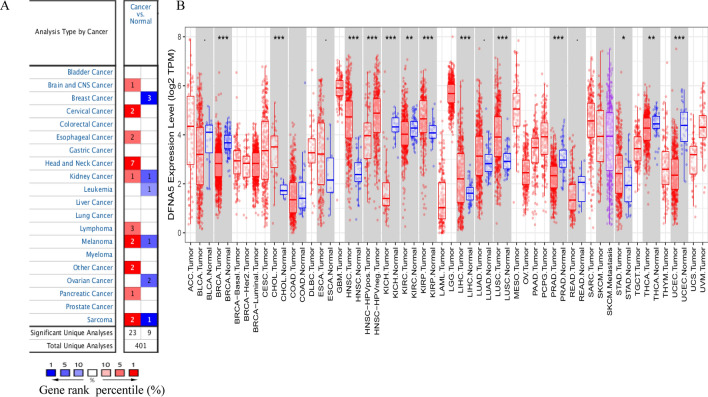
DFNA5 expression levels in diverse types of human cancers. **A** DFNA5 expression levels in diverse cancers relative to normal tissues in the Oncomine database. **B** Human DFNA5 expression levels in adjacent normal tissues and related tumour types in the TCGA database were obtained by TIMER (*P < 0.05, **P < 0.01, ***P < 0.001)

The differences in DFNA5 expression between tumours and normal tissues in diverse cancer types were analysed using the Oncomine database. This analysis showed that DFNA5 expression was higher in kidney cancer, lymphoma, pancreatic cancer, cervical cancer, head and neck cancer, melanoma, and sarcoma than in normal tissues (Fig. 1A).

To assess DFNA5 expression in diverse cancers, we investigated DFNA5 expression from RNA-seq data of diverse cancers in TCGA. The differential expression analysis between the tumour and adjacent normal tissues for DFNA5 of all TCGA tumours is displayed in Fig. 1B. DFNA5 expression was significantly lower in BRCA (breast invasive carcinoma), KICH (kidney chromophobe), PRAD (prostate adenocarcinoma), and UCEC (uterine corpus endometrial carcinoma) than in adjacent normal tissues. However, DFNA5 expression was significantly higher in LIHC (liver hepatocellular carcinoma), CHOL (cholangiocarcinoma), HNSC (head and neck cancer), LUSC (lung squamous cell carcinoma), STAD (stomach adenocarcinoma) and THCA (thyroid carcinoma) than in adjacent normal tissues.

### Prognostic potential of DFNA5 in cancers

**Fig. 2:**
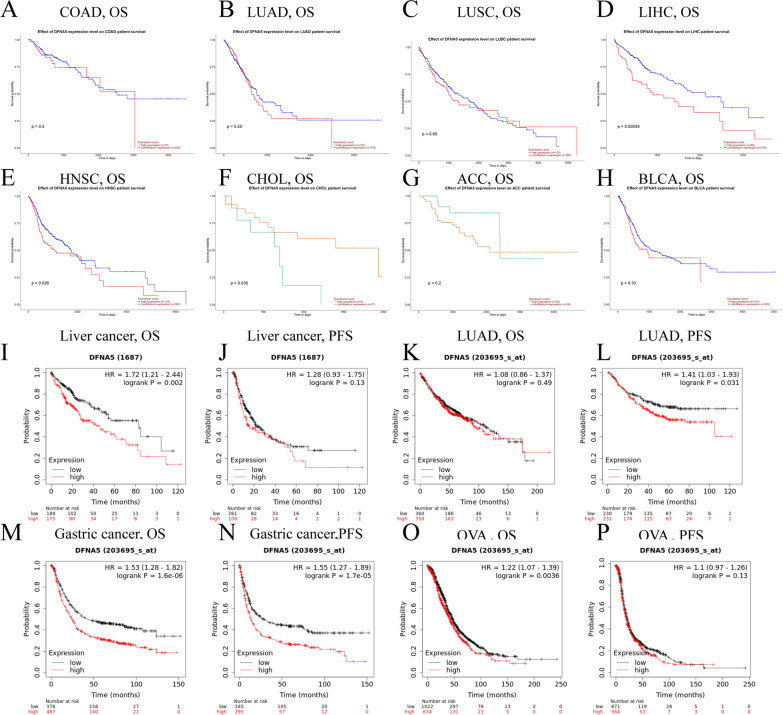
Kaplan–Meier survival curves comparing with high and low expression of DFNA5 in different types of cancer in the UALCAN (A–H) and Kaplan–Meier plotter databases (I–P). **A**–**H** Survival curves of OS in COAD, LUAD, LUSC, LIHC, HNSC, CHOL, ACC, BLCA. **I**, **J** Survival curves of OS and DFS in the liver cancer cohort. **K**, **L** High LUAD expression was correlated with poor PFS in the LUAD and survival curves of OS in the LUAD. **M**, **N** OS and PFS survival curves of gastric cancer. **O**, **P** OS and PFS of OVA. *OS* overall survival, *PFS* Progression Free Survival

We explored the relationship between DFNA5 expression and prognosis in cancer patients. The relationship of DFNA5 expression to overall survival was assessed by UALCAN. The correlations of DFNA5 expression with prognosis in diverse cancers analysed by UALCAN are presented in Fig. 2, Additional file 1: Figure S1. DFNA5 expression was significantly correlated with the prognosis in 5 types of cancers, including HNSC, THYM, CHOL, LIHC, and KIRC (Fig. 2, Additional file 1: Figure S1). Therefore, high DFNA5 expression may be an independent risk factor for a poor prognosis in these types of cancer. The Kaplan–Meier plotter database was used to investigate the potential prognostic value of DFNA5 in diverse cancers based on Affymetrix microarrays. Interestingly, a poor prognosis in gastric (OS HR = 1.53, 95% CI = 1.28 to 1.82, P = 1.6e-06; PFS HR = 1.55, 95% CI = 1.27 to 1.89, P = 1.7e-05) and LIHC (OS HR = 1.72, 95% CI = 1.21 to 2.44, P = 0.002; PFS HR = 1.28, 95% CI = 0.93 to 1.75, P = 0.13) was shown to correlate with higher DFNA5 expression. However, DFNA5 expression had less influence on OVA (Fig. 2) and showed a very weak correlation in LUAD (Fig. 2). These results suggest that DFNA5 expression has an impact on the prognosis of LIHC and gastric cancer (Fig. 2). Thus, we have identified the potential prognostic value of DFNA5 in given types of cancers and showed that decreased and increased DFNA5 expression has different potential prognostic value in diverse cancers.

### DFNA5 expression is correlated with immune infiltration level in colon, liver and lung cancers

**Fig. 3:**
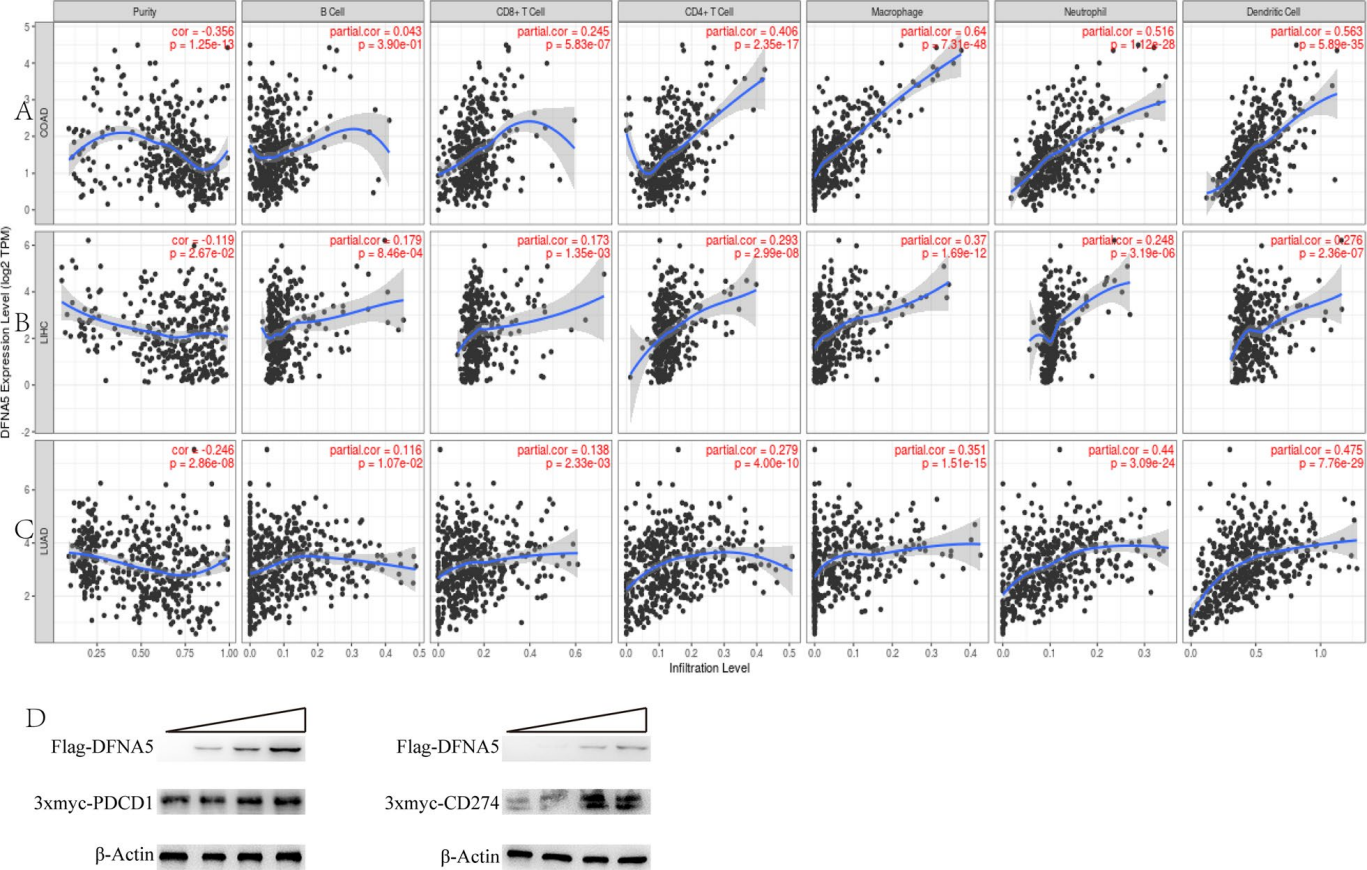
Correlation of DFNA5 expression with tumour-infiltrating lymphocytes in COAD (colon adenocarcinoma), LIHC (hepatocellular carcinoma), and LUAD (lung adenocarcinoma). **A** DFNA5 expression is significantly negatively related to tumour purity and has significant positive correlations with infiltrating levels of CD8 + T cells, CD4 + T cells, macrophages, neutrophils, and dendritic cells in COAD but not B cells. **B** DFNA5 expression has significant correlations with tumour purity and infiltrating levels of B cells, CD8 + T cells, CD4 + T cells, macrophages, neutrophils, and dendritic cells in LIHC. **C** DFNA5 expression is significantly negatively related to tumour purity and has significant positive correlations with infiltrating levels of B cells, CD8 + T cells, CD4 + T cells, macrophages, neutrophils, and dendritic cells in LUAD. **D** The increased content of Flag-DFNA5 plasmids co-transfected with 3x-myc-PDCD1 or 3x-myc-CD274 plasmid, the expression levels of FLAG, 3x-myc and β-actin were detected by western blot. The band grey value of the rightmost well of 3x-myc-PDCD1 or 3x-myc-CD274 relative to the corresponding beta-actin were set at 1.0. The other treatments were compared with them

Lymphocytes in tumour sites are an independent predictor of survival in specific cancers. We explored whether DFNA5 expression was correlated with the enrichment of tumour-infiltrating lymphocytes in diverse cancers. We evaluated the relationship of DFNA5 expression to the enrichment of immune cells infiltration in forty tumor types by Tumor Immune Estimation Resource. The data display that the expression levels of DFNA5 were pronounced association with tumour purity in Twenty-five tumor types. Also, the expression levels of DFNA5 were pronounced association with CD8 + T cells in fourteen tumor types, B cell in fourteen tumor types, macrophages in eighteen tumor types, neutrophils in twenty tumor types, CD4 + T cells in nineteen tumor types and dendritic cells in thirty-one tumor types (Fig. 3).

DFNA5 expression and tumour-infiltrating lymphocytes have significant correlations in diverse cancers. Tumour purity is a key element that impacts the analysis of tumour-infiltrating lymphocytes using genomic approaches, and GEPIA and TIMER have almost common data in TCGA. Interestingly, we discovered that the DFNA5 expression level was correlated with high immune infiltration and a worse prognosis in LIHC. The DFNA5 expression level had significant positive correlations with the infiltrating levels of B cells (r = 0.179, P = 8.46e−4), CD8 + T cells (r = 0.173, P = 1.35e−3), CD4 + T cells (r = 0.293, P = 2.99e−8), macrophages (r = 0.37, P = 1.69e−12), neutrophils (r = 0.248, P = 3.19e−6), and DCs (r = 0.276, P = 2.36e−7) in LIHC (Fig. 3B). Similarly, there were positive correlations with infiltrating levels of CD8 + T cells (r = 0.245, P = 5.83e−7), CD4 + T cells (r = 0.406, P = 2.35e−17), macrophages (r = 0.64, P = 7.31e−48), neutrophils (r = 0.516, P = 1.12e−28), and DCs (r = 0.563, P = 5.89e−35) in COAD (Fig. 3A). In addition, DFNA5 expression had pronounced association with tumour purity and enrichment of immune cells, including B cells, CD8 + T cells, macrophages, CD4 + T cells, dendritic cells, neutrophils in LUAD (Fig. 3C). Nevertheless, DFNA5 expression had no pronounced association with tumour purity and enrichment of immune cells, including B cells, macrophages, neutrophils, CD8 + T cells, CD4 + T cells, or dendritic cells in HNSC (Additional file 1: Figure S2). These data indicate that DFNA5 exerts key influences on enrichment of immune cells in lung cancers, liver and colon. 3x-myc-PDCD1 expression and 3x-myc-CD274 expression were detected by western blot. 3x-myc-PDCD1 expression or 3x-myc-CD274 expression were increased when co-transfected with increased concentration of Flag-DFNA5 plasmids (Fig. 3D).

### Correlation analysis between DFNA5 expression and immune marker sets

**Fig. 4:**
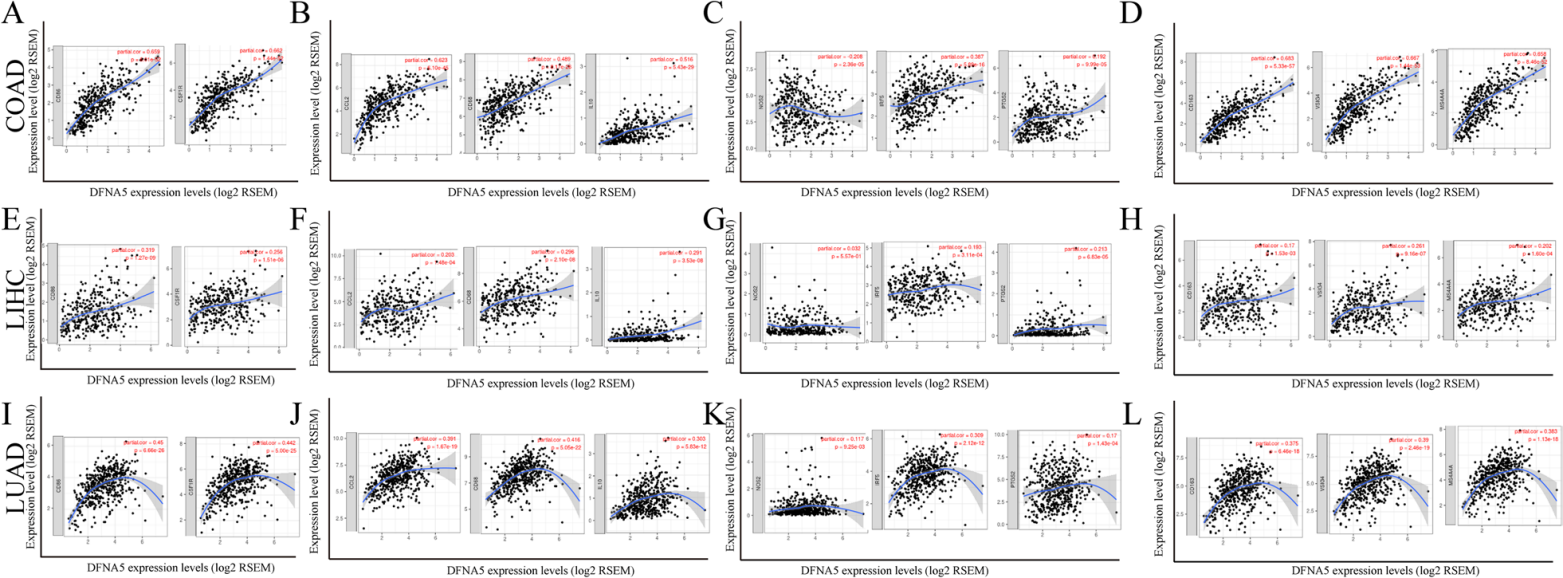
DFNA5 expression was correlated with macrophage polarization in COAD, LIHC and LUAD. The markers consist of CD86 and CSF1R of monocytes; CCL2, CD68, and IL10 of TAMs (tumour-associated macrophages); NOS2, IRF5, and PTGS2 of M1 macrophages; and CD163, VSIG4, and MS4A4A of M2 macrophages. **A**–**D** Scatter diagram of relationships between DFNA5 expression and gene markers of monocytes (**A**), TAMs (**B**), and M1 (**C**) and M2 macrophages (**D**) in COAD. **E**–**H** Scatter diagram of relationships between DFNA5 expression and gene markers of monocytes (**E**), TAMs (**F**), and M1 (**G**) and M2 macrophages (**H**) in LIHC. **I**–**L** Scatter diagram of relavances between DFNA5 expression and gene markers of monocytes (**I**), TAMs (**J**), and M1 (**K**) and M2 macrophages (**L**) in LUAD

To explore the correlation between DFNA5 and multiple tumour-infiltrating lymphocytes, we investigated the relationships between DFNA5 and marker sets of different immune cells of COAD, LIHC and LUAD in the GEPIA and TIMER databases. We determined the relationships of DFNA5 expression with marker genes of diverse immune cells, including CD8 + T cells, T cells (general), B cells, monocytes, TAMs, M1 and M2 macrophages, and neutrophils in COAD, LIHC and LUAD (Table 1; Fig. 4A–L). We also explored diverse functional T cells, such as exhausted T cells and Tregs. After the correlation adjustment by purity, we found that the DFNA5 expression level was significantly correlated with most marker sets of diverse immune cells in COAD, LIHC and LUAD. The DFNA5 expression level was significantly correlated with 27 gene markers in COAD, 27 gene markers in LIHC and 26 gene markers in LUAD (Table 1). Interestingly, we discovered that the expression levels of most marker sets of T cells, monocytes, TAMs, and M2 macrophages had strong correlations with DFNA5 expression in COAD, LIHC and LUAD (Table 1). Furthermore, chemokine (C–C motif) ligand (CCL)-2, CD68, and IL10 of TAMs and CD163, VSIG4 and MS4A4A of the M2 phenotype were significantly correlated with DFNA expression in COAD, LIHC and LUAD (Fig. 4A–L). Then, we performed an analysis of the correlation between DFNA5 expression and gene markers of monocytes and TAMs in the GEPIA database, including COAD, LIHC and LUAD. Correlation data between DFNA5 and markers of monocytes and TAMs were similar to those in TIMER (Table 2). The above results indicate that DFNA5 may induce macrophage polarization in COAD, LIHC and LUAD. T cell exhaustion is essential for immune escape [23, 24]. We also discovered significant correlations between DFNA5 and marker genes of Tregs and T cell exhaustion, such as FOXP3, CCR8, STAT5B, TGFb, CD274, PD-1, CTLA4, LAG3, and TIM-3 (Table 1). Interestingly, PDCD1, as a key gene that induces T cell exhaustion, has a strong positive correlation with DFNA5 expression, indicating that high DFNA5 expression plays a key role in PDCD1 mediating T cell exhaustion. Therefore, the above results indicate that DFNA5 is positively correlated with tumour-infiltrating lymphocytes in COAD, LIHC and LUAD. Furthermore, DFNA5 plays a key role in immune escape in the colon, liver and lung cancer microenvironment. The expression of PDCD1 and CD274 increase with increases in DFNA5 expression**.**

**Table 2:** Correlation analysis between DFNA5 and relate genes and markers of monocyte and macrophages in GEPIA

Description	Gene markers	COAD				LIHC				LUAD			
		Tumor		Nomal		Tumor		Nomal		Tumor		Nomal	
		R	P	R	P	R	P	R	P	R	P	R	P
Monocyte	CD86	0.74	***	− 0.15	0.34	0.33	***	0.41	*	0.23	***	0.25	0.057
	CD115 (CSF1R)	0.78	***	− 0.032	0.84	0.37	*	0.29	***	0.26	***	0.33	*
TAM	CCL2	0.65	***	0.19	0.24	0.13	0.016	0.44	*	0.24	*	− 0.06	0.65
	CD68	0.67	***	0.51	***	0.31	***	0.46	***	0.17	***	0.22	0.1
	IL10	0.45	***	− 0.069	0.67	0.26	***	0.51	***	0.2	***	0.33	*
M1 Macrophage	INOS (NOS2)-0.13		0.033	− 0.1	0.52	− 0.033	0.53	0.45	***	0.0074	0.087	− 0.13	0.33
	IRF5	0.3	0.033	0.09	0.58	0.17	*	0.26	0.063	0.16	***	0.29	0.024
	COX2 (PTGS2)0.24		***	0.0068	0.97	0.15	*	0.41	*	0.076	0.095	− 0.11	0.39
M2 Macrophage	CD163	0.61	***	− 0.036	0.82	0.22	***	0.25	0.082	0.2	***	0.27	0.037
	VSIG4	0.76	***	− 0.029	0.86	0.23	***	0.25	0.084	0.19	***	− 0.039	0.77
	MS4A4A	0.73	***	− 0.11	0.51	0.26	***	0.39	*	0.21	***	0.17	0.19

*LUAD* lung adenocarcinoma, *LIHC* liver hepatocellular carcinoma, *COAD* colon adenocarcinoma, *TAM* tumor-associated macrophage. Normal, correlation analysis in normal tissue of TCGA. Tumor, correlation analysis in tumor tissue of TCGA. ***P < 0.0001,**P < 0.001, *P < 0.01

### GO and KEGG enrichment analysis of chemokine and immune related genes interacted with DFNA5

**Fig. 5:**
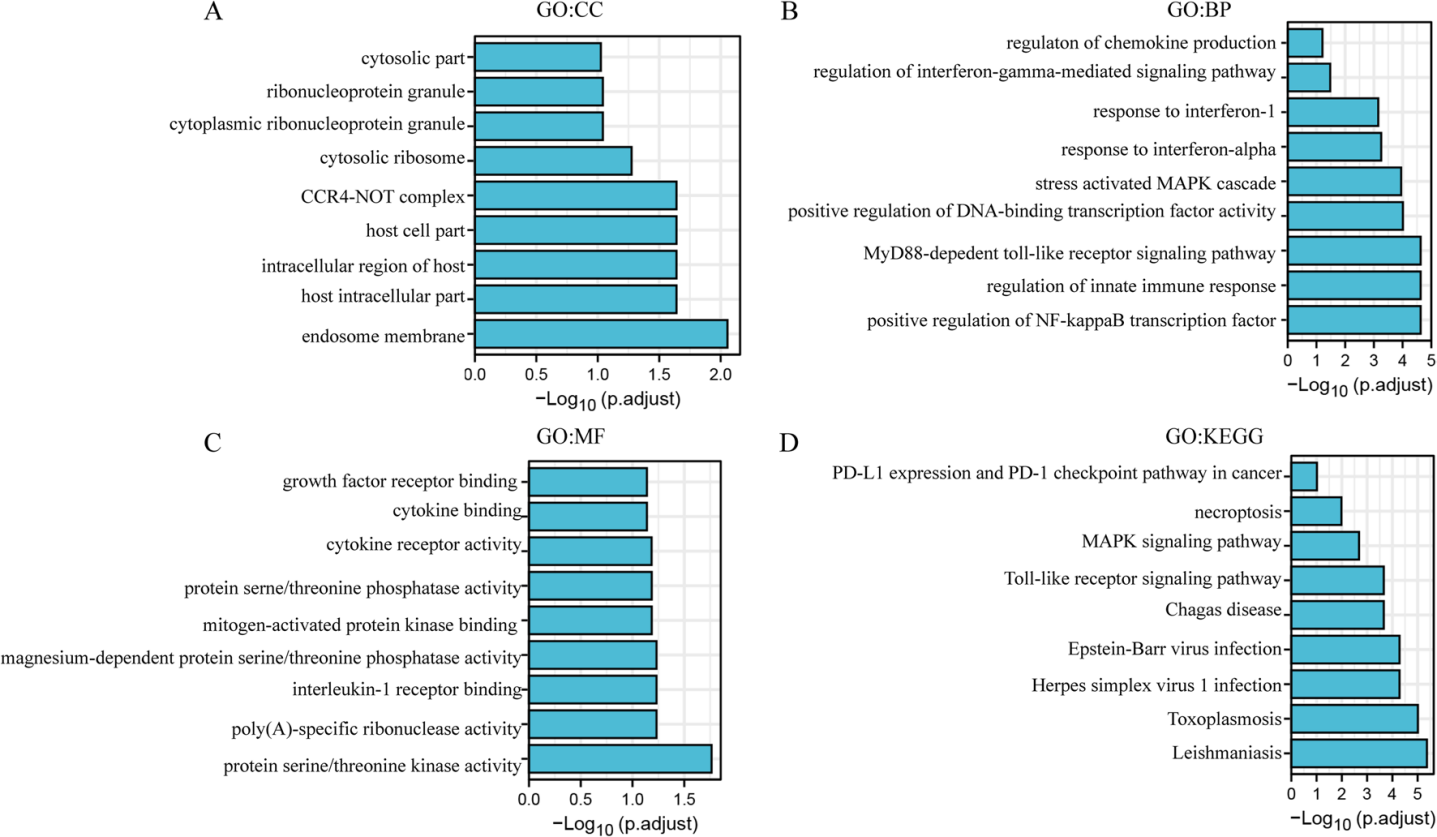
GO and KEGG enrichment analysis of chemokine and immune related Genes interacted with DFNA5 (DAVID). **A** BP, **B** CC, **C** MF, **D** KEGG

Protein–protein interaction is the basis of cell life and the whole biological field. Traditional methods were hardly preyed the instantaneous or weak interactions with target protein. TurboID system can explore the instantaneous or weak interactions with target protein. In this study, we screened the chemokine and immune related proteins interacted with DFNA5 using TurboID system. Chemokines can attract the immune cells to tumor sites and immune related proteins associate with antitumor effects. The screened proteins were existed in cytosolic part, ribosome, endosome membrane, cytoplasmic ribonucleoprotein granule (Fig. 5A). IFIT3, IRAK1 and CNOT7 could perform cellular response to type I interferon (Fig. 5B). IFIT3, EIF2AK2 could perform in response to interferon-alpha. IRAK1, TAB1, IRAK4 could exert in response to interleukin-1. EIF2AK2 could also exert in regulation of chemokine production. IRAK1, TAB1, IRAK4 could also exert in positive regulation of innate immune response. IFNGR1 could regulate the activity of cytokine receptor (Fig. 5C). In the KEGG analysis, only IFNGR1 was directly related to the functions of PD-L1 expression and PD-1 checkpoint pathway (Fig. 5D), which may explain the results of Fig. 3D.

## Discussion

DFNA5 was originally studied as a gene linked to deafness. Then, the vital role of DFNA5 in pyroptosis was discovered. DFNA5 is cleaved by caspase-3 in response to some chemotherapeutic drugs that could induce pyroptosis [14].

DFNA5 has been extensively studied, the role of DFNA5 in immune cell infiltration is poorly understood [15]. Here, the relation of prognosis in patients with diverse cancer types to DFNA5 expression levels is illustrated. Elevated expression levels of DFNA5 is related to a worse prognosis in LIHC, HNSC, THYM, CHOL, and KIRC. Furthermore, the data indicate that in liver, colon and lung cancers, diverse immune markers and immune infiltration levels are related to the levels of DFNA5 expression. The novel functions of DFNA5 in immune system are introduced to this study, and suggests it may be a potential marker in cancer.

Datasets of Oncomine and UALCAN were used to analyze DFNA5 expression and prognostic level in patients with diverse cancer types. The gene expression of normal tissues was different from diverse cancer types, we analyzed DFNA5 expression in Oncomine database and it was shown that was elevatedly expressed in pancreatic cancer, kidney cancer, lymphoma, cervical cancer, head and neck cancer, melanoma and sarcoma. However, DFNA5 expression levels in TCGA data in comparison to normal adjacent tissues were elevated in HNSC, LIHC, CHOL, LUSC, STAD and THCA but decreased in UCEC, PRAD, KICH and BRCA. Increased DFNA5 expression based on analysing the TCGA database indicated that it was related to an unfavourable prognosis in HNSC, THYM, CHOL, LIHC, and KIRC, while DFNA5 expression levels had no significant effects in other cancer types. Furthermore, a higher expression level of DFNA5 based on Kaplan–Meier Plotter indicated that it was related to an unfavourable prognosis in gastric, ovarian and liver cancers. In brief, these analysis indicate that DFNA5 may be used as a biomarker for prognosis in THYM, HNSC, CHOL, LIHC, KIRC, and OVA.

One of key aspect of analysis is that DFNA5 expression is relate to diverse lymphocytes infiltration in cancer. The date indicate that DFNA5 expression levels are positively related to the infiltrative level of CD8 + T cells, macrophages, neutrophils, CD4 + T cells and dendritic cells in COAD, LIHC and LUAD. Furthermore, the relation of immune cells markers to DFNA5 expression indicates DFNA5 in the regulation of tumour immunology in COAD, LIHC and LUAD. M1 macrophages gene markers including INOS (NOS2), indicated no positive relation to DFNA5 expression in COAD and LIHC, but M2 macrophage markers including MS4A4A, VSIG4 and CD163, indicated positive relationships in COAD, LIHC and LUAD. These results reveal the potential polarization role of DFNA5 in tumour-associated macrophages (TAMs). In addition, the increase in DFNA5 expression was positive correlation with the expression of Tregs (FOXP3, CCR8, and STAT5B) in COAD, LIHC and LUAD, which proved that DFNA5 has the potential to trigger Tregs. Furthermore, T cell exhaustion markers, including CD274, TIM-3, LAG3, CTLA4, and PD-1, which induce T cell exhaustion [6, 25–29], were increased in COAD, LUAD and LIHC. TIM3, PDCD1 and CD274, crucial surface proteins on exhausted T cells, were highly correlated with DFNA5 expression in COAD, LIHC and LUAD. The coexpression of DFNA5 and PDCD1 and CD274 indicated that DFNA5 could increase the expression levels of PDCD1 and CD274. The above relations may indicate that DFNA5 has a potential action to T cells in LIHC, COAD and LUAD. In brief, the data indicate that DFNA5 exert a key role in the regulation and recruitment of immune cells in LIHC, COAD and LUAD.

TurboID systems were used to identify instant or weak interaction proteins conveniently. Traditional methods are usually performed to investigate the interactive proteins with strong force, while it may be easy to omit the key proteins. In this research, we mainly focus on chemokines, interferon and immune checkpoint related proteins to investigate the role of DFNA5. IFIT3, IRAK1, CNOT7, EIF2AK2, TAB1, IRAK4, IFNGR1 can interactive with DFNA5, which may relate to promote immune cells infiltration and immune exhaustion. Chemokine and immune related proteins interact with DFNA5. EIF2AK2 could also exert in regulation of chemokine production. DFNA5 may regulate immune infiltration via EIF2AK2. IFNGR1 was related to the functions of PD-L1 expression and PD-1 checkpoint pathway, which may explain DFNA5 could regulate the PD-L1 and PD-1 expression levels and may improve antitumor therapy in combination to immune checkpoint inhibitors. Furthermore, Recent studies provide possible mechanisms that explain why DFNA5 expression correlates with immune infiltration and a poor prognosis. DFNA5 overexpression can cause significant enhancement of the secretion levels of many inflammatory factors, including interleukin MIP1α, MIP1β8, and IP-10, in the tumour tissue after chemotherapy treatment. These chemokines exert an important role in T-cell infiltration. Tumour cells can secrete many types of chemokines to recruit immune cells into tumour sites. In addition, DFNA activation in macrophages can also recruit NK cells into tumour tissue. However, while DFNA5 expression correlates with a poor prognosis in several cancers, it has no correlation with the prognosis of most cancers. This may be due to T cell exhaustion marker overexpression that triggers immune cells insensitive to tumour cells.

In summary, increased DFNA5 expression correlates with a poor prognosis in liver cancers and increased immune infiltration levels of CD8 + T cells, CD4 + T cells, macrophages, neutrophils and DCs of multiple cancers, especially in colon, liver and lung cancers. DFNA5 expression potentially induces polarity changes in tumour-associated macrophages, Tregs and T cell exhaustion. Therefore, DFNA5 likely plays an important role in immune cell infiltration and is a new potential target for cancer immunotherapy in the clinic.

## Supplementary Information

**Additional file 1: Figure S1**. Survival curves of diverse cancers in Kaplan–Meier. **Figure S2**. (A) DFNA5 expression is significantly negatively related to tumor purity and has significant positive correlations with infiltrating levels of CD8+ T cells.

## Data Availability

All remaining data are available within the article and supplementary files, or available from the authors upon request.

## Abbreviations

PD-1: Programmed death-1
PD-L1: Programmed death ligand-1
CTLA4: Cytotoxic T lymphocyte associated antigen 4
TAMs: Tumour-associated macrophages
