# Supplementary material for: DFNA5 regulates immune cells infiltration and exhaustion
Source: Cancer Cell Int. 2022 Mar 5;22:107. doi: 10.1186/s12935-022-02487-0 (PMC8897971; doi:10.1186/s12935-022-02487-0)
Supplement: Supplementary file 1 — Additional file 1: Figure S1. Survival curves of diverse cancers in Kaplan–Meier. Figure S2. (A) DFNA5 expression is significantly negatively related to tumor purity and has significant positive correlations with infiltrating levels of CD8+ T cells. [file 12935_2022_2487_MOESM1_ESM.docx]

**Additional Material**


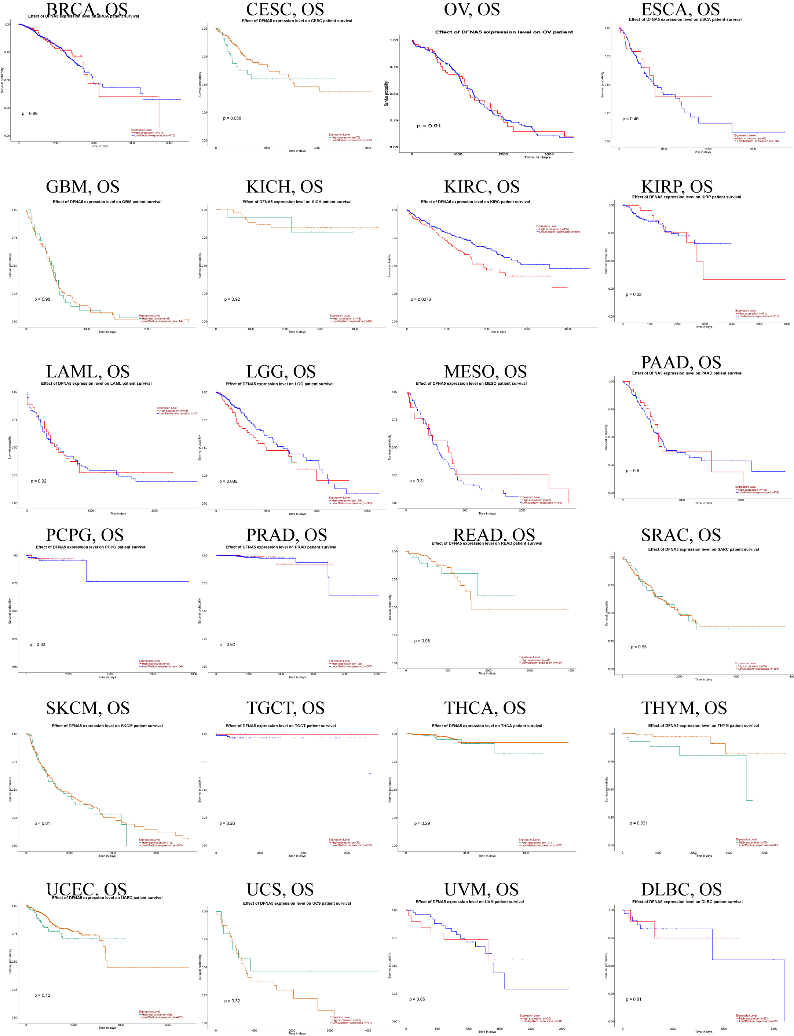


S1. Survival curves of diverse cancers in Kaplan-Meier


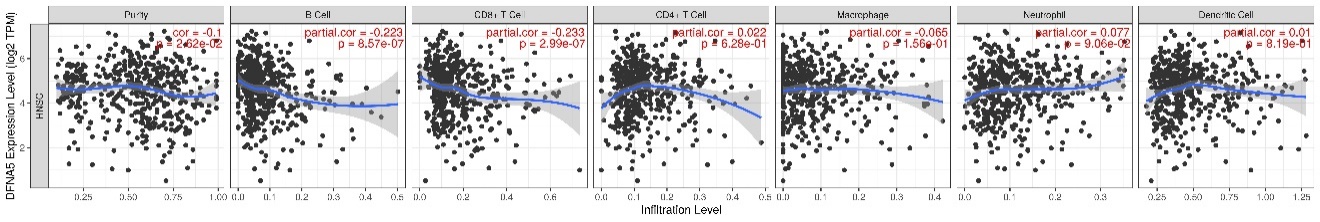


S2. (A) DFNA5 expression is significantly negatively related to tumor purity and has significant positive correlations with infiltrating levels of CD8+ T cells .
